# Aesthetic Outcome of a Case of Orbital Floor Fracture Treated Using a Retroseptal Transconjunctival Approach

**DOI:** 10.7759/cureus.4063

**Published:** 2019-02-13

**Authors:** Kalarikkal Mukundan Harish, Guruprasad Tulasidas, Babu Arthanari, James Antony Bhagat

**Affiliations:** 1 Oral and Maxillofacial Surgery, Ragas Dental College and Hospital, Chennai, IND; 2 Oral and Maxillofacial Surgery, SRM Dental College, Chennai, IND; 3 Dental Surgery, Southern Railway Hospital, Chennai, IND; 4 Oral and Maxillofacial Surgery, Adhiparasakthi Dental College, Chennai, IND

**Keywords:** orbital floor fracture, retroseptal transconjuctival approach, scarless approach

## Abstract

The orbital floor is a bone structure frequently involved in orbital fractures. Various methods have been documented to approach the orbital floor and infraorbital rim. Traditionally, transcutaneous approaches like infraorbital, subciliary, and subtarsal have been employed to access the orbital floor and infraorbital rim. A significant amount of complications including a visible, prominent scar, eyelid abnormalities like ectropion, lower lid retraction, and increased scleral show result from these transcutaneous approaches. To overcome these complications, the transconjunctival approach has been preferred recently. However, the transconjunctival approach has been associated with rare complications like entropion, synechia, or trichiasis. In the present article, we report a case of orbital floor fracture treated using a retroseptal transconjunctival approach. We intend to evaluate the aesthetic outcome of a case of orbital floor fracture treated using a retroseptal transconjunctival approach.

## Introduction

Lang initially described an orbital blow fracture in 1889 as the mechanism by which an impact to the eyeball was transposed as mechanical energy to the orbital walls, causing them to fracture [1,2]. The various indications for surgery in an orbital fracture were described by Gassner et al. as follows: 1) Fractures involving one half or more of the orbital floor and/or medial wall; 2) Computed tomography (CT) evidence of orbital soft tissue entrapment; 3) Diplopia and ocular motility limitation within thirty degrees of primary position; 4) Enophthalmos of more than 2 mm; and 5) Hypesthesia of the infraorbital nerve territory [3]. Despite executing proper surgical technique and achieving a successful anatomic reconstruction of the orbit, a few complications like enophthalmos, diplopia, and hypesthesia of the infraorbital nerve territory were encountered at a long-term follow-up [4-6]. The various surgical accesses to the orbit include the following: 1) A transorbital approach using a skin or conjunctival incision; 2) A transantral approach; and 3) An endoscopic endonasal approach [7]. The various transcutaneous approaches, namely, the infraorbital incision, subtarsal incision, and subciliary incision, have demonstrated a significant rate of complications. However, transconjunctival incision has comparatively few complications like entropion and scleral show. The preseptal transconjunctival approach is more cumbersome whereas a retroseptal transconjunctival approach has a relatively direct approach to the orbital rim and floor. The transconjunctival approach has recently gained popular acceptance among maxillofacial surgeons, although it requires appropriate training in orbital surgery and meticulous execution of the technique. We present a case of orbital zygomaticomaxillary complex fracture where the orbital floor and infraorbital rim were fixed via a retroseptal transconjunctival approach and the postoperative aesthetic outcome of the same was evaluated.

## Case presentation

**Figure 1:**
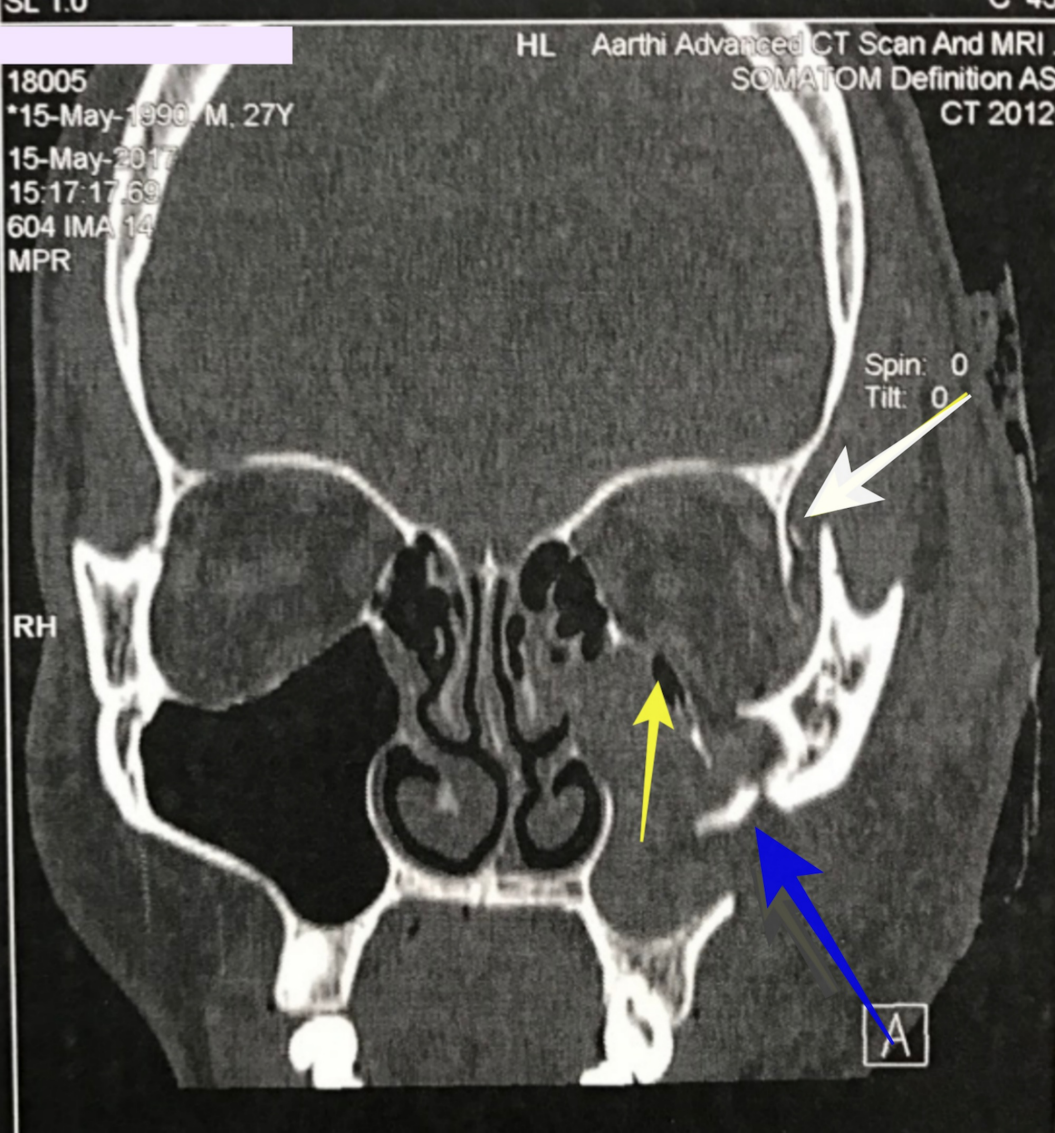
Computed tomography scan (coronal view) demonstrating fracture at left front zygomatic region (white arrow), orbital floor involving the infraorbital rim (yellow arrow) along with fracture of the left zygomatic maxillary buttress region (blue arrow).

**Figure 2:**
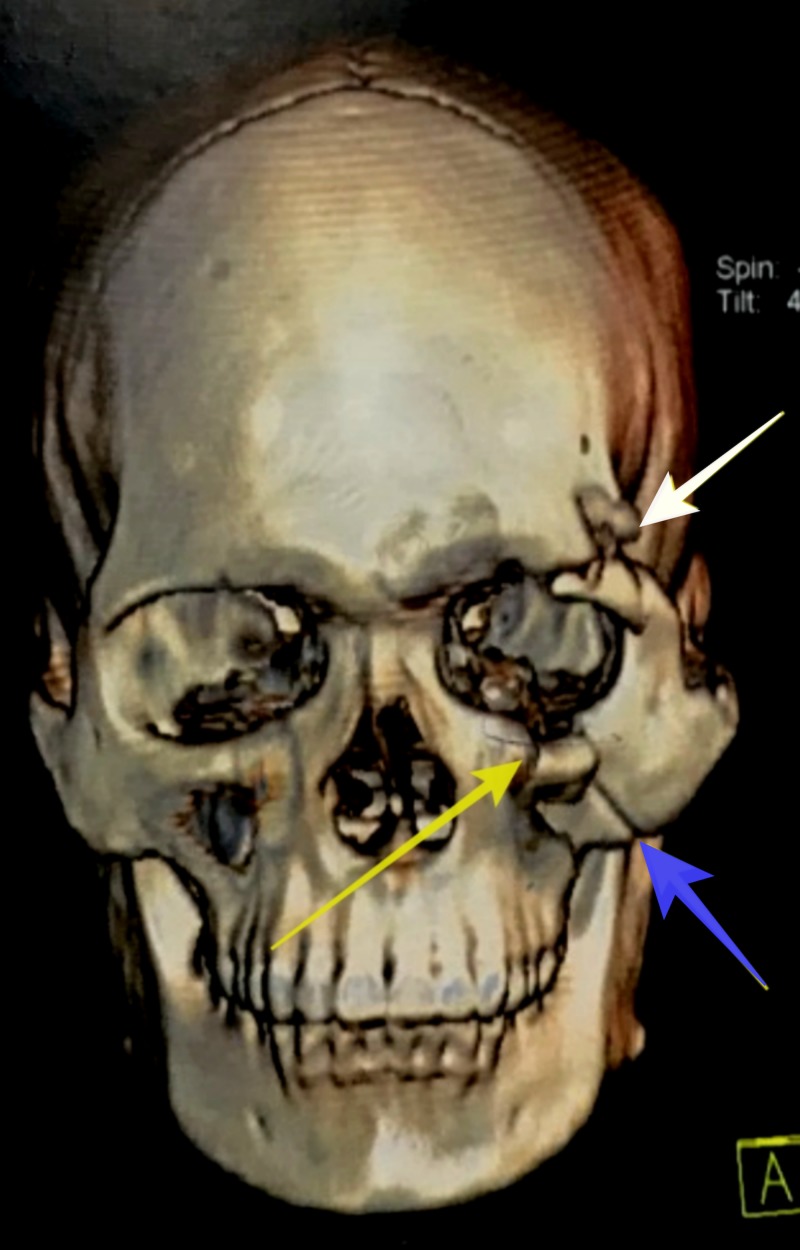
Virtual three-dimensional reconstruction of computed tomography scan demonstrating fracture at left front zygomatic region (white arrow), left zygomatic maxillary buttress (blue arrow), left infra orbital rim (yellow arrow).

A 27-year-old male patient reported to Southern Railway Headquarters Hospital, Perambur, Chennai, with an alleged history of trauma to the face. He had a loss of consciousness after injury. He had no history of vomiting or posttraumatic amnesia. He underwent a neurological examination, and any possibilities of intracranial hemorrhage or cranial injury were ruled out. He was declared fit to undergo surgical repair of the facial fractures under general anesthesia. He underwent a detailed ophthalmic investigation by an ophthalmologist and was documented as exhibiting normal visual acuity, an absence of relative afferent pupillary defect, and normal eye movements, thus excluding injury to the globe or optic nerve. On extraoral examination, we noted circumorbital edema around the left eye and subconjunctival hemorrhage in the left eye. There was a laceration of the forehead around the frontozygomatic area on the left side. The patient had abrasions over the left zygomatic region. There were no other associated lacerations or abrasions on the face. On intraoral examination, occlusion was intact, and no fracture or mobility of any teeth was noted. The mouth opening was 38 mm. We noted normal temporomandibular jaw movements both in protrusion and lateral movements. There were tenderness and crepitation in the left frontozygomatic region and at the intraoral zygomaticomaxillary buttress region. On radiological investigation, a CT scan revealed a fracture at the left frontozygomatic region, the orbital floor involving the infraorbital rim, along with a fracture of the left zygomaticomaxillary buttress region (Figures 1, 2).

**Figure 3:**
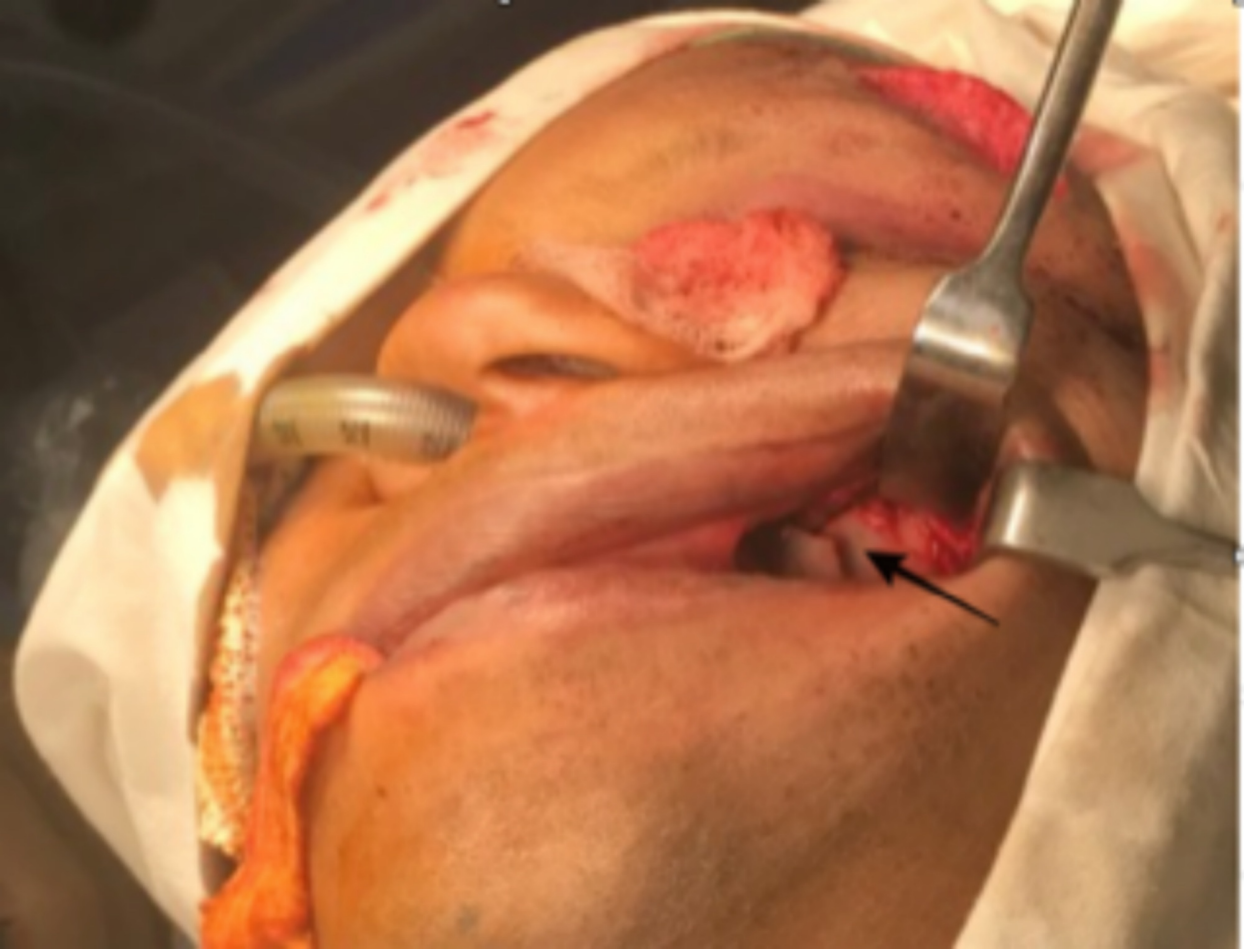
Fracture at left zygomatic maxillary buttress region.

**Figure 4:**
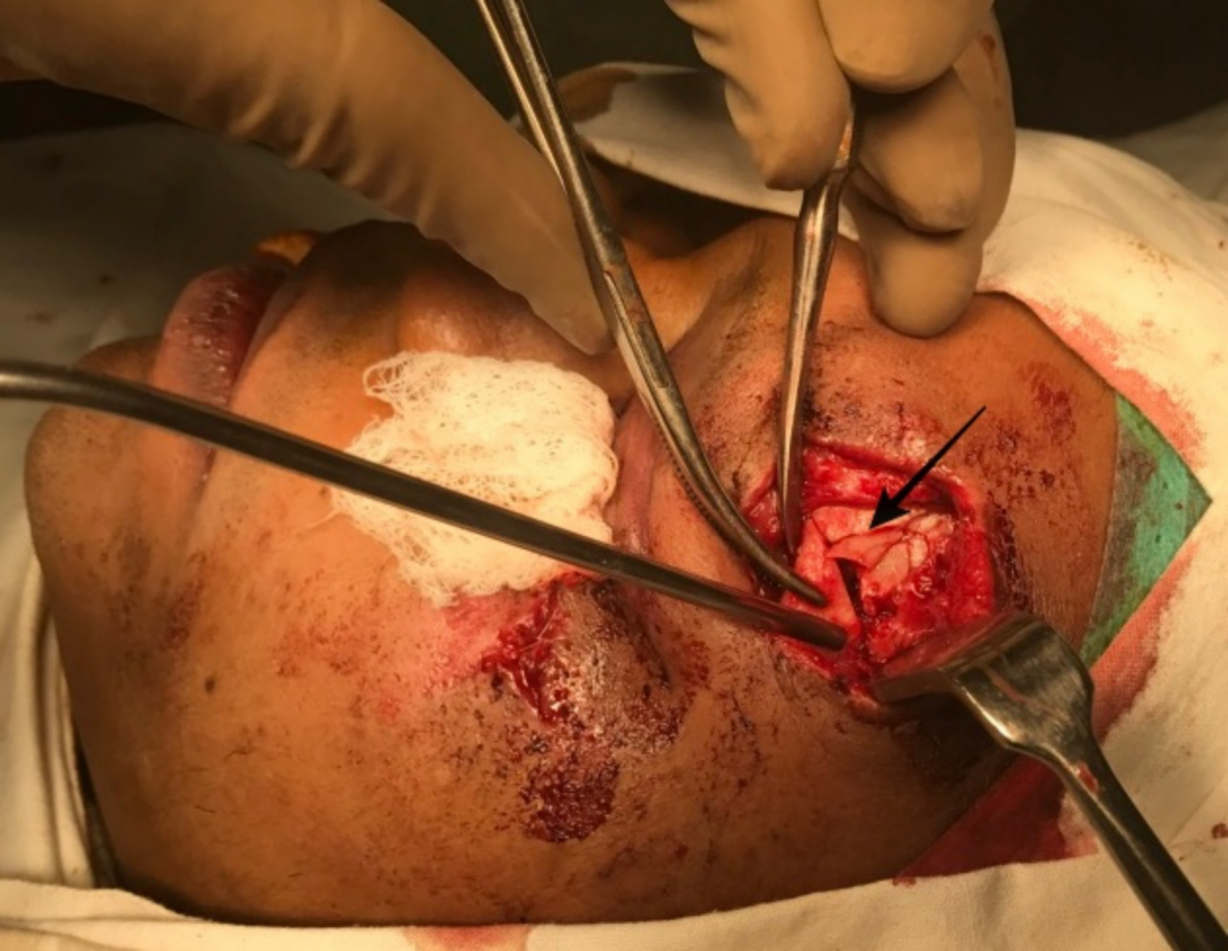
Exposure of the fracture at left front zygomatic region via the existing laceration.

**Figure 5:**
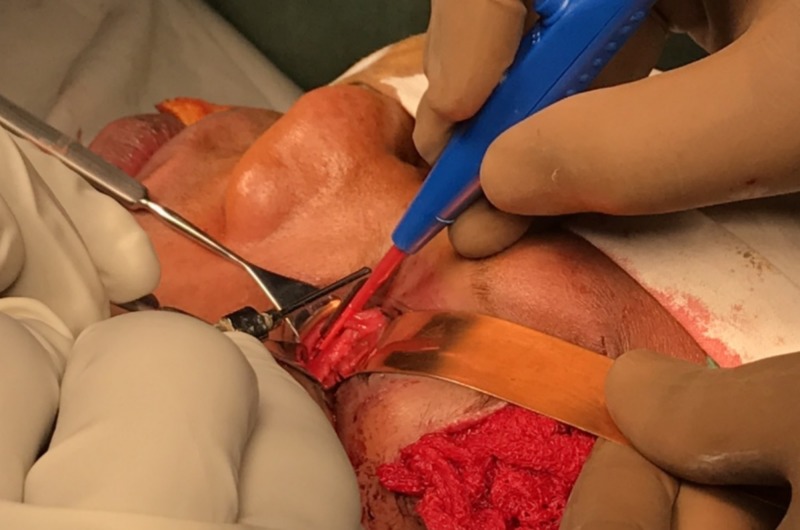
Execution of retroseptal transconjunctival incision.

**Figure 6:**
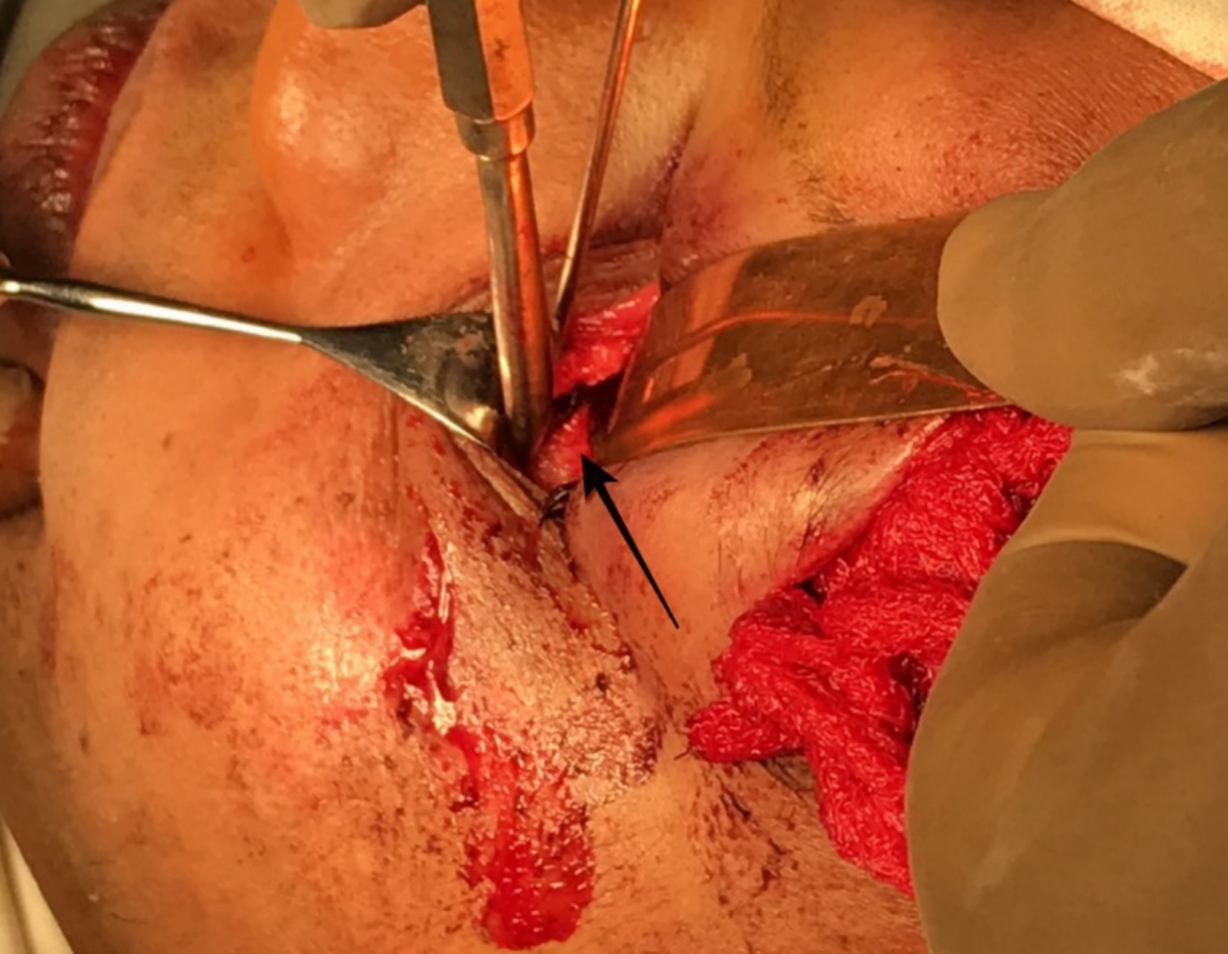
Exposure of the fractured orbital floor.

**Figure 7:**
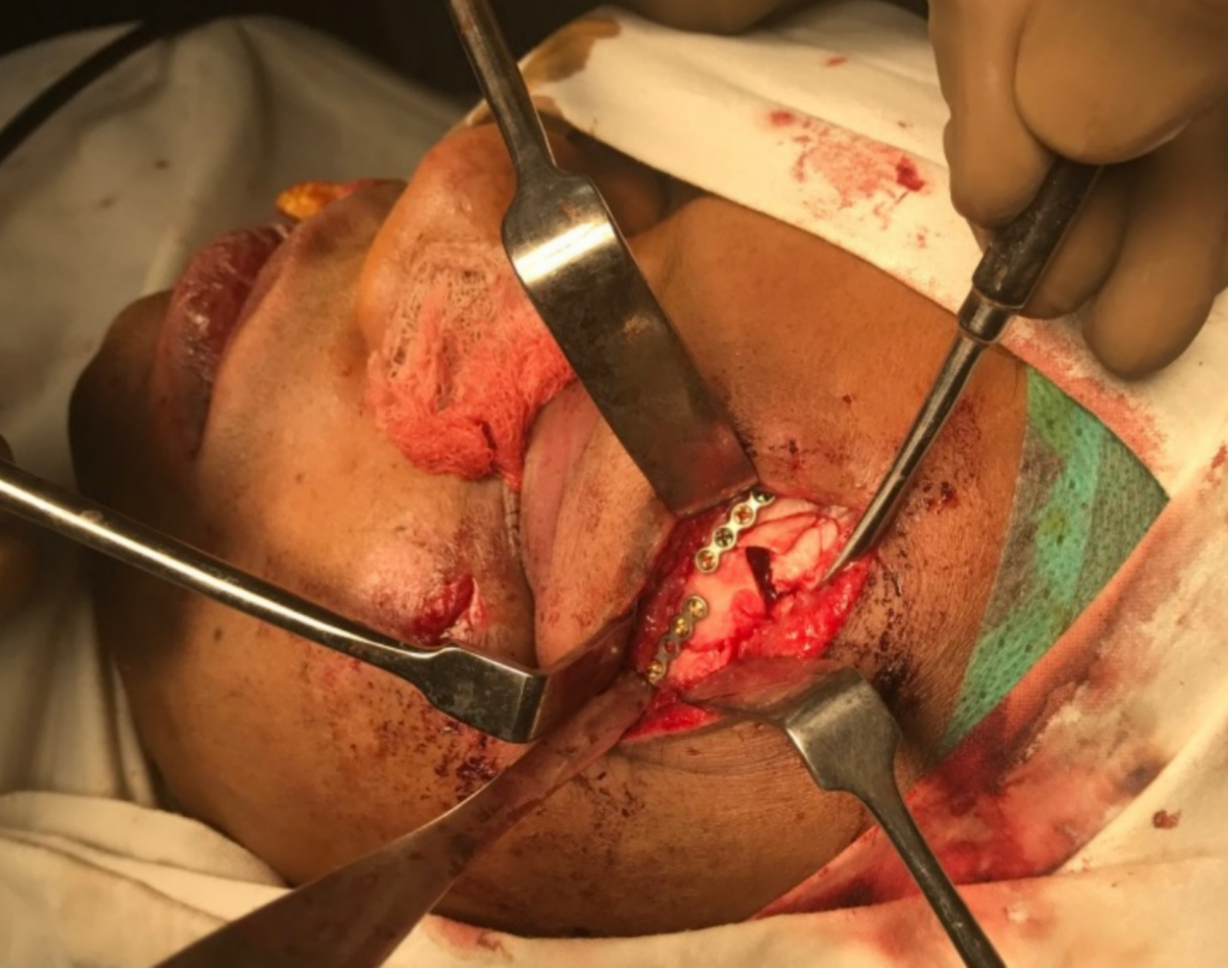
Miniplate fixation at the frontozygomatic region.

**Figure 8:**
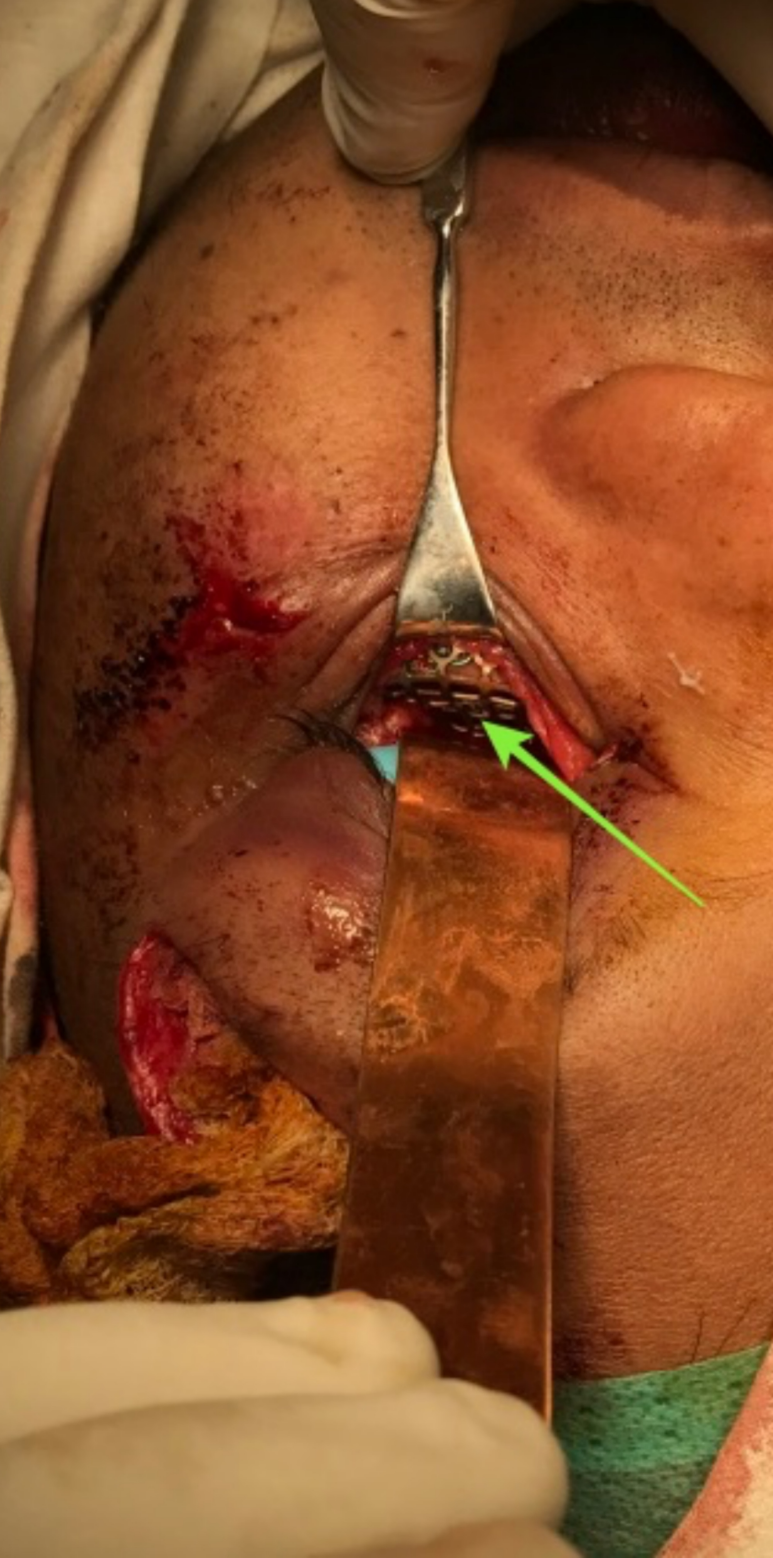
Placement of orbital mesh.

​​​​​​An open reduction and internal fixation of the fractured left orbital zygomaticomaxillary complex was planned under general anesthesia, three days post-trauma. Under general anesthesia, the left zygomaticomaxillary buttress was surgically exposed via a vestibular incision after performing a subperiosteal dissection (Figure 3). The fracture at the left frontozygomatic region was exposed via the already existing laceration (Figure 4). A retroseptal transconjunctival approach was used to access the infraorbital rim and orbital floor. A Desmarres retractor was used to retract the lower eyelid. To prevent corneal abrasions or tearing and to protect the eyeball, a corneal shield was used. The inferior fornix was held with toothed tissue-holding forceps, and an incision was placed into the palpebral conjunctiva using Colorado tip (Stryker CMF, Chicago, IL, USA) electrocautery between the lowermost point of the eyelid and the inferior fornix (Figure 5). Scissors were used to locate the inferior orbital rim, and the periorbital was reached. A subperiosteal dissection was done along the orbital floor to expose the fractured orbital floor using a malleable retractor in a dog paddle maneuver (Figure 6). The dissection was continued until a stable posterior ledge of bone was reached on the orbital floor. After achieving surgical exposure of the fracture sites, the reduction was achieved by employing Rowe’s zygomatic elevator. After ensuring adequate reduction, the following fixation was carried out: 1) Fixation of frontozygomatic suture region was performed using two four-hole titanium plates (Synthes) without gap (1.5 mm × 6 mm; Figure 7); 2) Fixation of left zygomaticomaxillary buttress using one straight four-hole plate without gap (2 mm × 6 mm); 3) Fixation of orbital floor and infraorbital rim using a preformed orbital titanium mesh (Universal Orbital plate, 0.4 mm Synthes Maxillofacial [Paoli, PA]), that was fixed on to infraorbital rim using 1.5-mm × 6-mm titanium screws (Figure 8).

**Figure 9:**
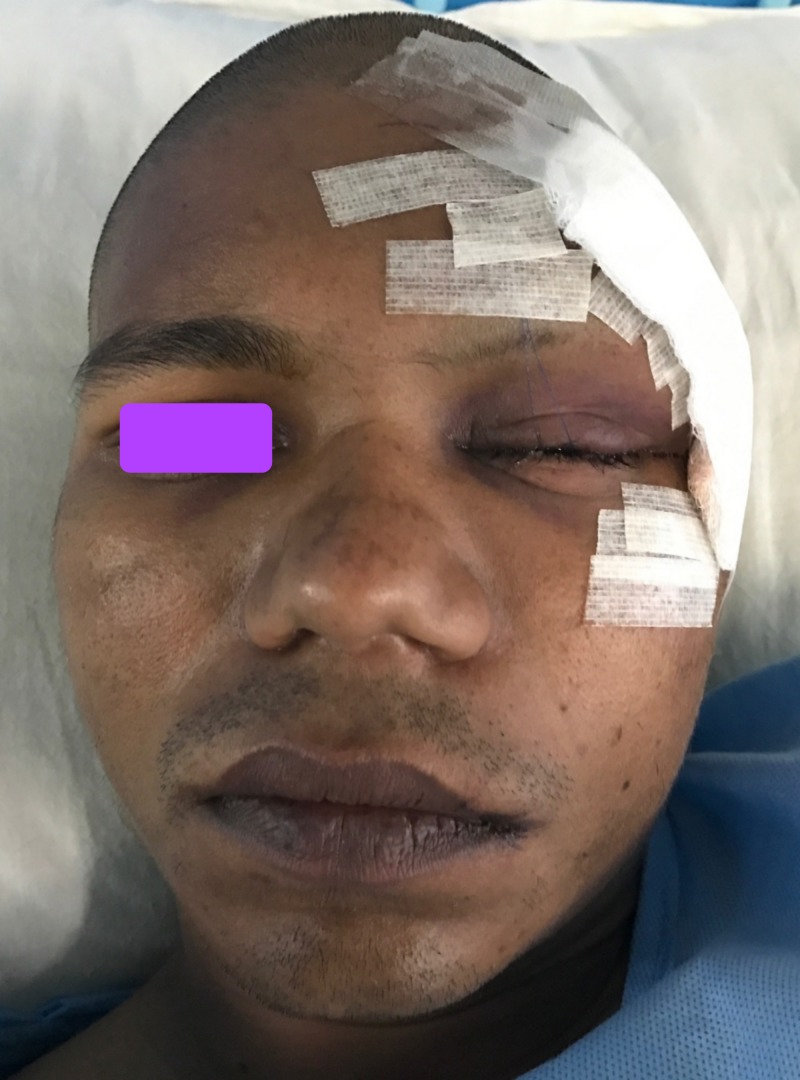
Frost suspension suture.

The retroseptal transconjunctival incision was closed using 5-0 Vicryl buried sutures, and a Frost suture was suspended from the lower tarsal plate for seven days postoperatively (Figure 9).

The wound at the frontozygomatic region was closed in two layers, namely the muscle layer using 4-0 Vicryl simple interrupted sutures, and the skin layer using 6-0 Prolene simple interrupted sutures. The intraoral zygomaticomaxillary buttress suture was closed using 4-0 Vicryl simple interrupted sutures. The patient was prescribed postoperative topical antibiotic drops (ciprofloxacin) and eye lubricants (carboxymethyl cellulose) for five days, along with cefotaxime and metronidazole intravenous antibiotics and diclofenac sodium intramuscular injection as an analgesic for five days.

## Discussion

The involvement of facial bones in road traffic accidents is constantly on the rise, which can be attributed to a lack of usage of helmets or seat belts. The face bears the brunt of injury in road traffic accidents. The prominence of the zygoma bone in the face makes it more vulnerable to injury in trauma involving the face. The zygoma bone is convex in shape and has a tetrapod articulation, namely [8]:

· The maxilla at the zygomaticomaxillary buttress and infraorbital rim

· The frontal bone at frontozygomatic suture

· The temporal bone forming the zygomatic arch

· The sphenoid bone at sphenozygomatic suture

**Figure 10:**
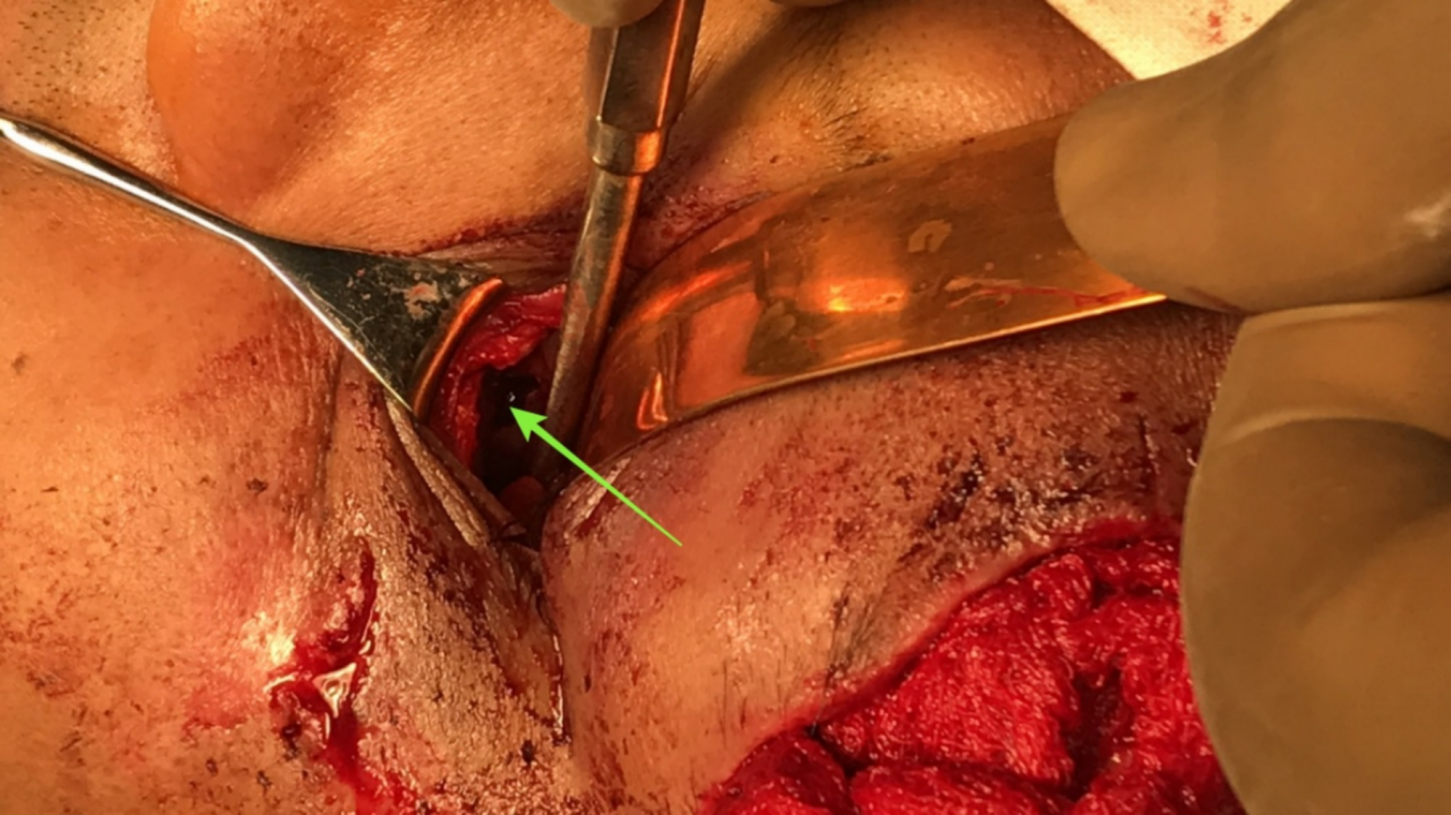
Exposure of the orbital floor via a retroseptal transconjunctival approach.

The precise anatomic reduction and fixation of orbital zygomaticomaxillary fracture are of prime importance in restoring the facial contour in all three dimensions of the face. In our case, the patient presented with diffuse swelling of the left orbital and zygoma region with an alleged history of trauma to the face. After performing a detailed clinical and radiological examination, an open reduction and internal fixation of the zygomatic and frontal bones were planned under general anesthesia, after obtaining ophthalmic and neurosurgery clearance. The zygomatic bone was fixed at three different sites of articulation, namely: 1) the zygomaticomaxillary buttress using an intraoral vestibular approach; 2) the infraorbital rim using a retroseptal transconjunctival approach; 3) the frontozygomatic suture using the existing laceration. The main goal in open reduction and internal fixation of a zygomaticomaxillary complex fracture is to establish a three-dimensional reduction and stable fixation adequately. After exposing the sites mentioned above, reduction of the fracture was carried out using Rowe’s zygomatic elevator. The most reliable method to evaluate the adequacy of reduction involves the direct inspection of the fracture reduction of the zygomaticosphenoid suture at the lateral orbital wall [9-12]. The zygomaticomaxillary buttress and infraorbital rim are considered other reliable sites to evaluate the adequacy of fracture reduction, whereas the frontozygomatic suture is not considered as a reliable site for visual judgment for fracture reduction [9,11,13]. In our case, we visually evaluated the zygomaticomaxillary buttress and infraorbital rim for adequacy of reduction. Once we were satisfied with the reduction, we proceeded with fixation of the fracture. A variety of transcutaneous incisions like infraorbital, subtarsal, and subciliary incisions have been described to access the infraorbital rim and the orbital floor. In addition to the visible scar, other complications like ectropion, scleral show, and lower lid retraction have been well documented. According to Werther, the infraorbital incision is preferred for cases with marked edema that precludes the accurate placement of subciliary or subtarsal incisions [14]. According to Patel et al. and Appling et al., higher rates of scleral show and ectropion have been reported with subciliary incision than that of transconjunctival incision [15,16]. In our case, we preferred a retroseptal transconjunctival approach to access the infraorbital rim and the orbital floor, due to the advantage of direct access and scar-free nature of the incision. In this approach, the conjunctiva was dissected from behind the orbital septum down to the bony orbit (Figure 10).

**Figure 11:**
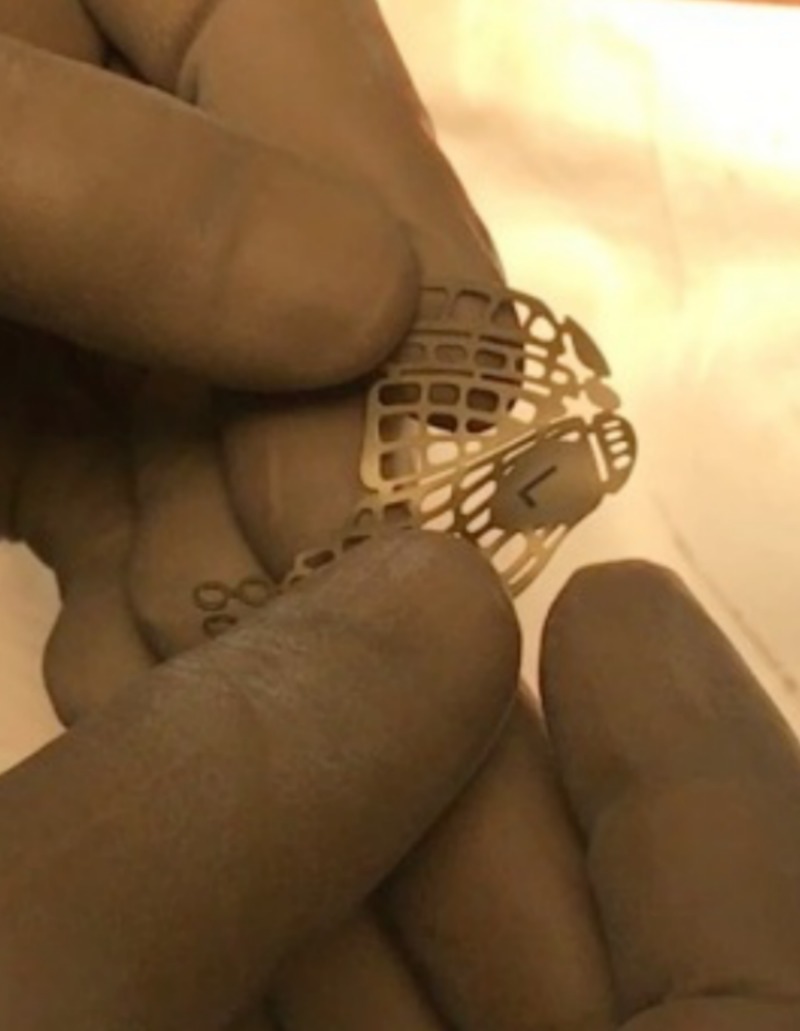
Preformed orbital mesh (DePuy Synthes, Zuchwil, Switzerland).

**Figure 12:**
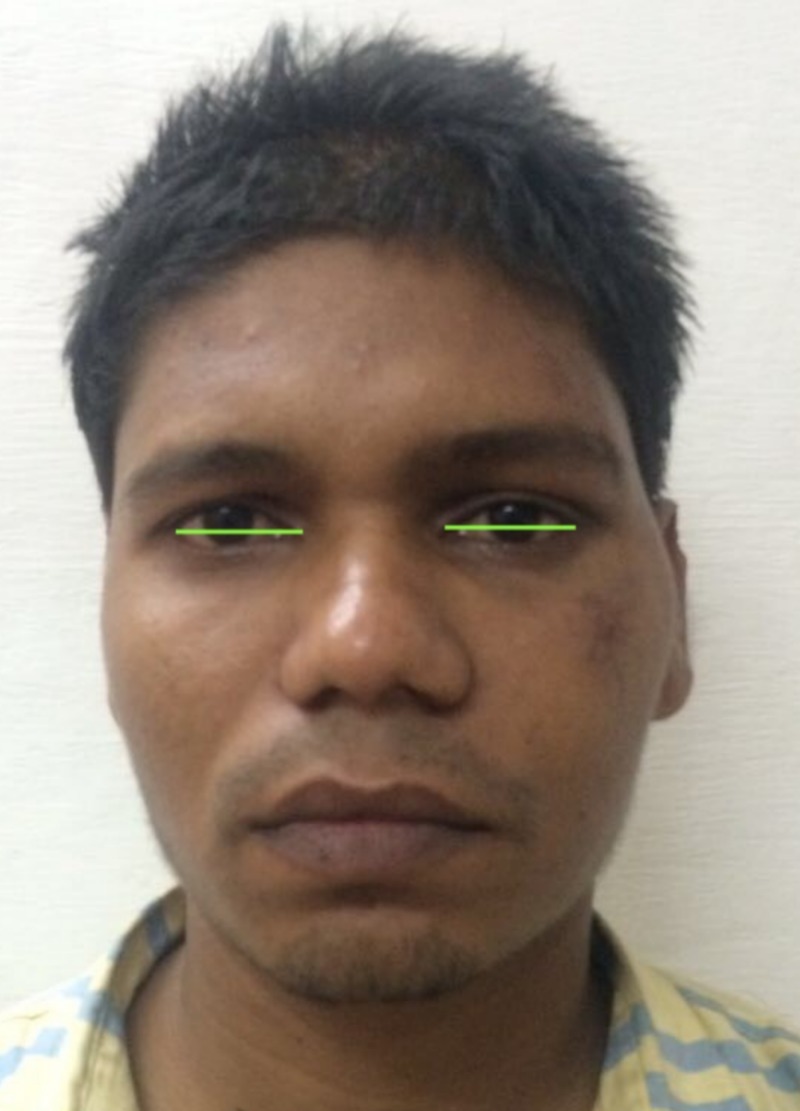
Image showing absence of canthal tilt.

**Figure 13:**
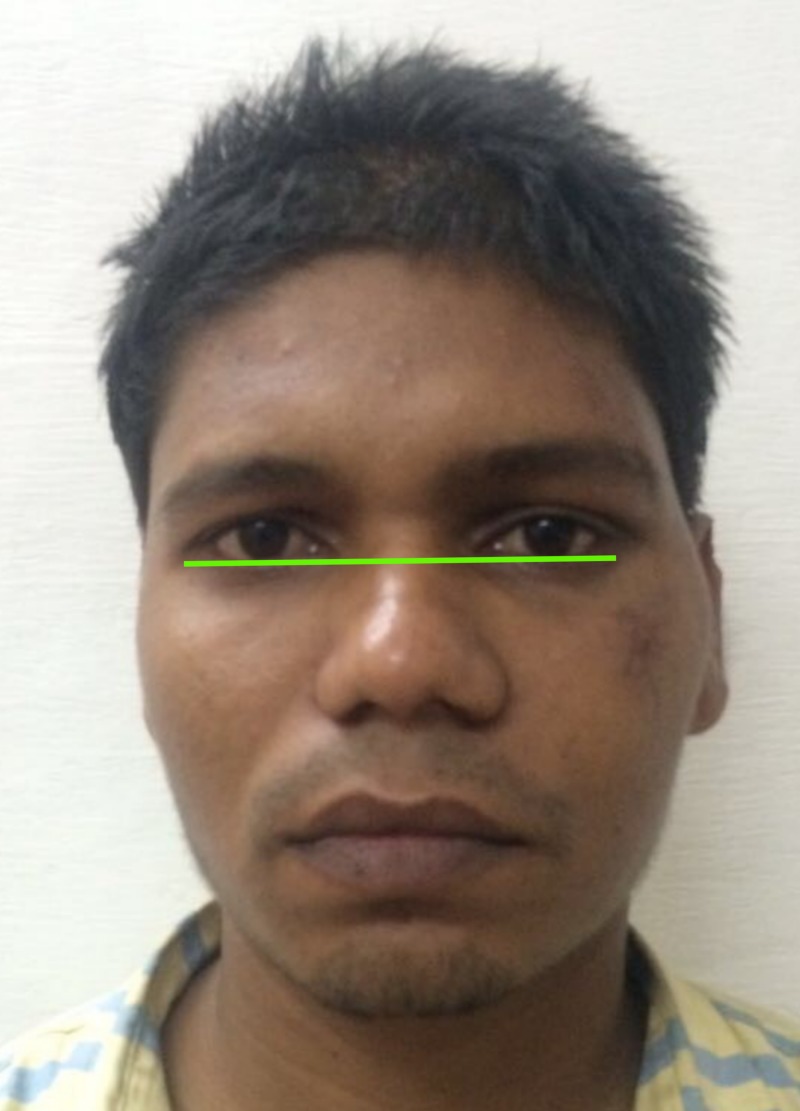
Image showing absence of scleral show.

**Figure 14:**
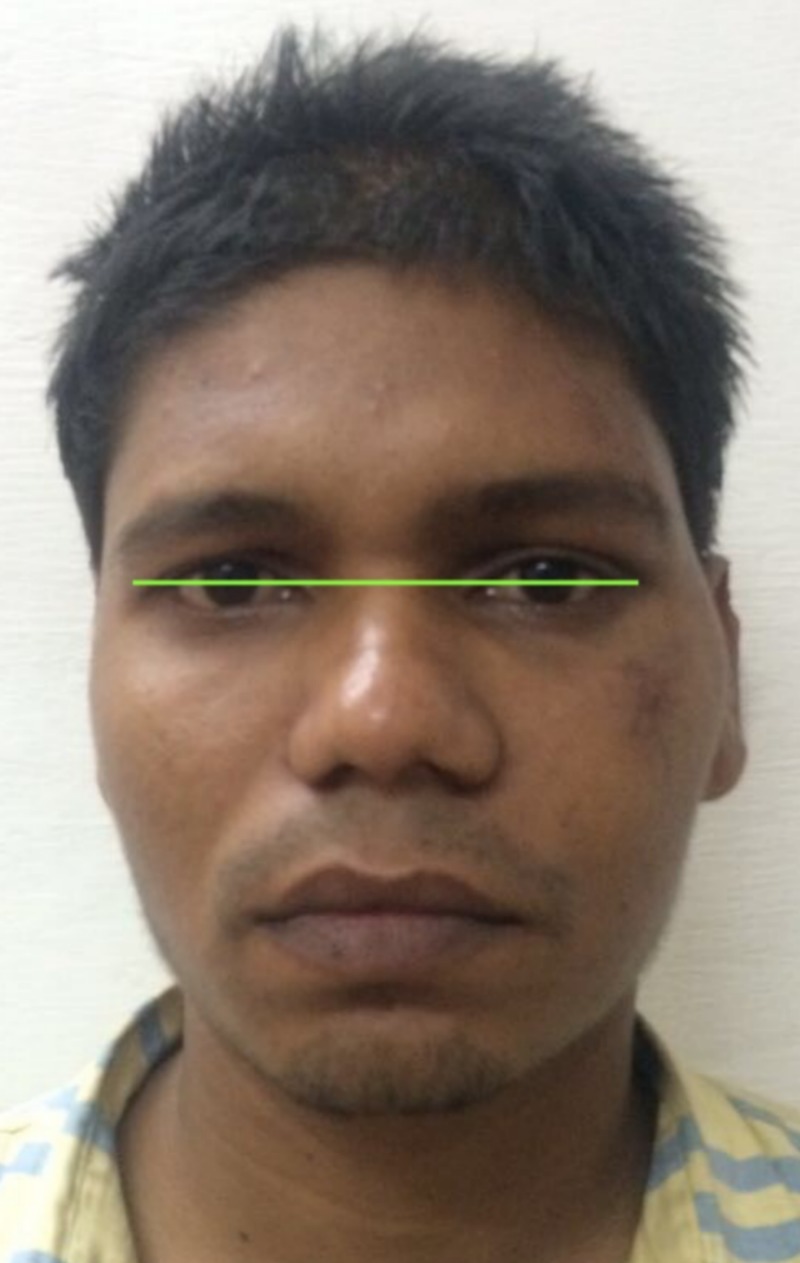
Image of patient eyes showing lack of difference in the pupillary level.

**Figure 15:**
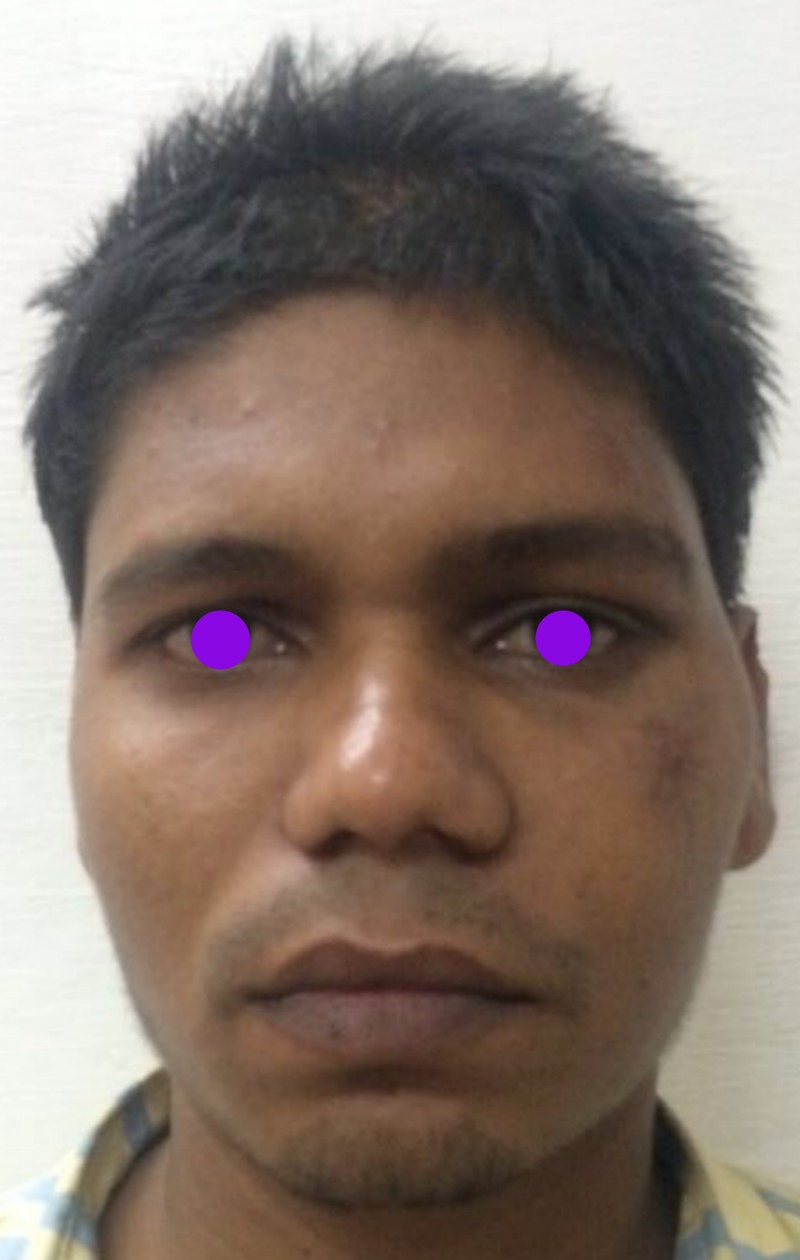
Image showing lack of excessive skin and muscle demonstrated by absence of redundant lower eyelid anterior lamella.

The lateral canthotomy along with a transconjunctival incision allows the periosteum to be elevated superiorly to repair the frontozygomatic suture. However, we did not perform a lateral canthotomy as the access to the frontozygomatic suture was gained through the existing laceration. In accordance with the study carried out by Holtmann et al., the average skin-to-fracture exposing time was five to eight minutes for the infraorbital rim and subtarsal incision, and the subciliary incision was executed in 15 minutes [17]. According to Santosh and Giraddi, the average time taken for exposing a fracture site using preseptal transconjunctival approach was 21 minutes [18]. In our case, the time taken for exposure of the infraorbital rim via a retroseptal transconjunctival approach was around six minutes, and another five minutes was taken to explore the orbital floor until a stable posterior bony ledge was verified. The orbital mesh was placed with the posterior margin of the mesh resting on a stable posterior ledge of bone, and anteriorly the mesh was fixed on the infraorbital rim. The purpose of the internal orbital reconstruction material is to isolate the orbital contents from the antrum or nasal cavity and to provide postoperative support sufficient to prevent enophthalmos. According to Haug et al., the weight of the combined internal orbital contents was 42.97 ± 4.05 g (range, 37.80 to 51.03 g). The universal orbital plate 0.4 mm (Synthes Maxillofacial [Paoli, PA]) used in our case for orbital floor reconstruction had a maximum load of 8.46 ± 5.34 kg and a maximum displacement of 6.14 ± 3.24 mm (Figure 11) [19]. Thus, the orbital mesh employed in our case would serve as an excellent source of orbital support taking the above-mentioned parameters into account. The fractures at the frontozygomatic region and adjacent frontal bone were fixed using the existing laceration as access. The patient was evaluated six months following surgery. At the follow-up evaluation, we found no signs of infection, pain or paresthesia at the operated sites. The aesthetic outcome of the surgery was evaluated by assessing the presence or absence of canthal tilt, scleral show, pupillary levels, and excessive skin muscle [20]. The canthal tilt was assessed using a line connecting the medial to lateral canthus. It was neutral (i.e., both the medial and lateral canthus were at the same level; Figure 12). Scleral show was present when the lower eyelid was inferior to the lower limbus at a forward gaze. There was an absence of scleral show in this case (Figure 13). The pupillary level was assessed taking the level of mid-pupil of both eyes, and both pupils were assessed at the same level (Figure 14). Excessive skin and muscle were defined by the presence of redundant lower eyelid anterior lamella, which was absent in our case (Figure 15).

## Conclusions

The employment of a transconjunctival retroseptal approach to access the infraorbital rim and orbital floor provides a highly aesthetic and satisfactory outcome postoperatively, provided the applied orbital anatomy is understood in detail and meticulous execution of the technique mentioned above is performed. We believe with a larger number of oral and maxillofacial surgeons preferring this technique, along with a lateral canthotomy, the transconjunctival retroseptal approach will become the major workhorse approach in the future to treat orbitozygomatic complex fractures.
